# Dietetics

**Published:** 1905-07-22

**Authors:** 


### DIETETICS.

(*Continued from page* 278.)

**Proteids in Diet.**—The present view as to the breaking up of proteids taken as food is, according to Halliburton,[5] that they, like fats and carbohydrates, must first be split up into simple substances, which explains why each animal can construct tissues peculiar to itself out of varied raw material. Proteids directly injected into the blood are apparently retained, the splitting up being carried on by tissue erepsins and similar intracellular ferments. Halliburton points out that the results of Chittenden's recent work, by which he showed that one half the amount of proteid usually considered necessary was enough to maintain health, are not conclusive, as no allowance had been made for reserve force to meet extra strain. Hutchison[6] points out that most practical dietaries approach nearer the old standard, and thinks mere nitrogenous equilibrium is not a satisfactory criterion of the sufficiency of the proteid ingested. Starling[7] holds similar views, dividing the total necessary nitrogen into that required for growth which could not be replaced by other substances, and that required for the production of energy which could be replaced by fats and carbohydrates. Seligman[8] notes that natives of New Guinea who live on a low nitrogen diet lack stamina, but Martin[9] believes that ordinary diet contains too much nitrogen (17 grams), most people only requiring 10 grams. This fault he says is exemplified in the rations served to the British army. In the Tropics, Wiley[10] shows that less proteid and less fatty food is required. Less heat is lost by radiation, the habits of the people are less active, and hence less tissue waste occurs. The natural food of indigenous peoples illustrates this, being composed largely of fruits, containing little proteid and fat, but much carbohydrate, especially sugar. The cocoa nut is the chief source of fat, while some variety of cassava provides the carbohydrate.

**Diet in Disease.**—In typhoid fever Thompson[11] recommends semi-solid food the day the temperature first reaches normal—this to include baked custard, junket, milk toast, light broths, soft-boiled eggs, blanc-mange, farina, boiled rice with beef juice. On the third day of convalescence he gives a scraped beef sandwich. He limits this treatment to mild cases without complications; it should not be pursued if there is much tympanites, diarrhœa, or recent severe hæmorrhage. He points out that milk itself is a solid food in the stomach, and is badly tolerated by some patients. Cases where nutrition fails rapidly and the temperature remains low (101°–102° F.), with the passage of curds in the stools and meteorism, are best treated by predigested milk, beef juice, white of egg, milk-sugar, orange juice, and gruels; even before the temperature is normal, semi-solid food may be given with advantage. When milk is very badly borne, it should be entirely stopped for two or three days, beef juice, egg white, and broths being substituted. Thompson does not think relapses are in the least influenced by treatment such as this. Peabody[12] does not wait for the temperature to become normal, but relies on the general condition and appetite as an indication for semi-solid food. He considers that eggs boiled for 20 or 30 minutes are more digestible than soft-boiled eggs. Robinson,[13] while agreeing in the main with Peabody, suggests modifying the milk with pancreatin and sodium bicarbonate to aid its digestion. Abbe notes that surgical cases often show a transient rise of temperature when first put on solid food, and thinks such a rise may also occur in typhoid, and need not indicate a true relapse. Janeway[14] mentions two cases in which a relapse followed solid food given 17 days after the temperature had become normal, but declines to say positively that this was a case of cause and effect. Thompson,[15] as the result of experiment, did not consider hard-boiled eggs so easily digested and absorbed as raw eggs, although they were more friable. In arthritis deformans Thompson advocates a light but very nutritious diet, with plenty of milk and red meat, similar to that often given in phthisis, and Gibney[16] is in the habit of allowing these patients to eat what they fancy most. Apart from acute nephritis, in which milk is universally admitted to be necessary and suitable, Thompson considers that the danger of eating meat in nephritis is overrated. In cases in which the only symptom is albuminuria he allows a moderate amount of red meat; in those who are also anæmic and weak, with perhaps a tendency to uræmia, meat is not indicated, but periods of vegetarian or infantile diet, alternating with a period in which proteid food may be allowed. With regard to fluids in cases of anarsarca, he thinks it dangerous to diminish fluids when there is a corresponding fall in the quantity of urine passed. In other cases a dry diet may lead to absorption of effused fluid, but here and in passive effusions this treatment is often disappointing. In cases of nephritis, complicated by other disease such as phthisis or diabetes, two points must be remembered — the most threatening disease must have precedence in matters of diet, and nutrition must as far as possible be maintained. Janeway restricts fluids where the pulse has a high tension, and the heart is weak and hypertrophied. In diabetes Thompson agrees with Hutchison that the individual and not the disease must be considered, and that no routine diet can be recommended. He considers gluten preparations unsatisfying and unreliable, and is inclined to prefer small quantities of toast or potato chips, according to the power of the patient to tolerate them. Well-developed cases require a rigid diet for a week or two, and then a measured amount of bread; mild cases require chiefly proteid diet with a moderate amount of fat and carbohydrate; and in very severe cases both proteids and carbohydrates should be restricted and large amounts of fat given. In lithæmia and arteriosclerosis he advises a week or ten days of vegetarian diet with 60 or 70 ounces of water per diem—two glasses on rising and going to bed and two before each meal. Open-air life, rest after meals, and moderate exercise are advisable. As improvement occurs the diet may be increased till at last small quantities of red meat are allowed once a day. In arteriosclerosis, fluids should be restricted when there is myocarditis or dilatation, but not if nephritis is present. Allan Starr[17] also advises vegetable diet for lithæmics with neurasthenia, for a week or two; but in organic nervous disease, such as locomotor ataxia, such a restriction of diet is often harmful. In an acute attack of gout milk, gruels, and Vichy water suffice; but in the intervals Thompson advises a reduction in the total amount of food taken, an increase in the consumption of water, the total exclusion of sugar and alcohol, and a minimum amount of red meat. A rigid vegetarian diet for a few weeks may be advantageous, but if too long continued produces anæmia and weakness. Janeway has recorded two cases of gout, in one of which the attacks were much more frequent while the patient was on rigid milk diet, and in the other the first acute seizure came on while the patient was under treatment for duodenal ulcer. The diet in rheumatism should be similar to that in gout, but Thompson thinks the results far less satisfactory. In children it is important not to restrict diet too much, owing to the necessity for providing for the growth of the body.

[5] Lancet, March 18, 1905, p. 716. [6] Ibid., p. 717. [7] Ibid. [8] Ibid. [9] Ibid. [10] Brit. Med. Jour. Epit., Dec. 3, 1904, No. 325. [11] Med. Rec., Dec. 10, 1904, p. 921. [12] Ibid., p. 954. [13] Ibid. [14] Ibid. [15] Ibid., p. 955. [16] Ibid. [17] Ibid.

*(To be concluded.)*

